# Failure to thrive as presentation in a patient with 22q11.2 microdeletion

**DOI:** 10.1186/s13052-016-0224-0

**Published:** 2016-02-11

**Authors:** Grazia Bossi, Chiara Gertosio, Cristina Meazza, Giovanni Farello, Mauro Bozzola

**Affiliations:** Paediatrics Department, Fondazione IRCCS Policlinico San Matteo, Pavia, Italy; Internal Medicine and Therapeutics Department, Paediatric and Adolescent Unit, University of Pavia, Fondazione IRCCS Policlinico San Matteo Piazzale C. Golgi 2, 27100 Pavia, Italy; Pediatric Unit, Department of Life Health and Environmental Sciences, University of L’Aquila, L’Aquila, Italy

**Keywords:** Failure to thrive, Growth hormone deficiency, Chromosome abnormalities

## Abstract

**Background:**

Abnormalities of chromosome 22q11, including deletions and translocations, have been described in association with different birth defects and malformations occurring in many combinations and degrees of severity.

**Case presentation:**

We describe the case of an 8 month-old infant with no dysmorphic signs who showed progressive postnatal growth failure and no chronic systemic diseases. We found a 22q11.2 microdeletion, inherited from the mother, suggesting the diagnosis of DiGeorge syndrome. The patient had an isolated growth hormone (GH) deficiency and a significant increase in linear growth during the first and the second year of GH therapy, and a recovery of weight was shown.

**Conclusions:**

Sometimes, in infants with growth failure a genetic analysis is strongly suggested, since chromosomal abnormalities may be present.

## Background

Abnormalities of chromosome 22q11, including deletions and translocations, have been described in association with different birth defects and malformations occurring in many combinations and degrees of severity [1]. In particular, deletion of chromosome 22q11.2 is causally related to DiGeorge syndrome (DGS) [1], velocardiofacial syndrome (VCFS) [2] and conotruncal anomaly face syndromes [3]. The prevalence of these 22q11 deletion syndromes (DS) is about 1/1,000 foetuses, according to the study of Grati et al. [4]. Although many patients are diagnosed before age 2 years, the diagnosis during childhood and even adulthood is not so rare, also due to changing of phenotypes associated with these syndromes. Although cardiac malformations and immunodeficiency are the cardinal manifestations [5], short stature is a common problem associated with 22q11.2 microdeletion syndrome [6], also in the absence of cardiac defects. Many studies reported growth retardation and short stature in children with DGS/VCFS [7, 8], and in some cases growth hormone deficiency (GHD) was also diagnosed [7].

Here, we describe a short infant who was investigated because of a progressive failure to thrive. After exclusion of chronic systemic diseases, a genetic analysis was requested although no particular syndromic signs were present and a diagnosis of DiGeorge syndrome was made.

## Case presentation

An 8 month-old girl, the only child of non-consanguineous healthy parents was referred to our Department because of failure to thrive (height: 64.3 cm, −2.12 standard deviation score (SDS); weight: 5,680 g, body mass index (BMI) 0.57 SDS according to Cole). She was born by caesarean section at the 39^th^ week for interruption of foetal growth, with a birth weight of 2,450 g (BMI −1.82 SDS) and length of 47 cm (−1.26 SDS). Apgar score was not available, but spontaneous breathing, without cyanosis and jaundice, was documented.

No increase in weight was observed after a period of adequate diet and supplementation of vitamins. Systemic diseases including hypothyroidism, skeletal dwarfism and malabsorption, in particular celiac disease, and common syndromes, such as Turner, were excluded.

Her progressive growth failure, which is typical of primitive dwarfism, led us to investigate the subject from a genetic point of view, although no dysmorphic signs were present. The comparative genomic hybridization array (array-CGH) showed a 17q12 duplication of approximately 692 kb, with no pathogenetic significance, and a 22q11.2 deletion of approximately 2.57 Mb. A diagnosis of 22q11.2DS was therefore made, in the absence of typical features of this syndrome [9]. In fact, thyroid-parathyroid ecocolordoppler and complete abdomen ultrasound were normal. The echocardiography showed the presence of patent foramen ovale with left to right shunt without hemodynamic significance. Both humoral and cellular immune system was found to be normal, confirming the absence of systemic infections. Finally, no otolaryngologic, immunologic and neurocognitive problems were reported. The mother showed the same deletion as her own child, notwithstanding normal phenotype and only some relatively modest learning and behavioural problems, such as mild cognitive deficit, borderline intellectual function, poor social skills and unipolar mood. She was therefore given genetic counselling, since she was interested in having another child.

**Fig. 1:**
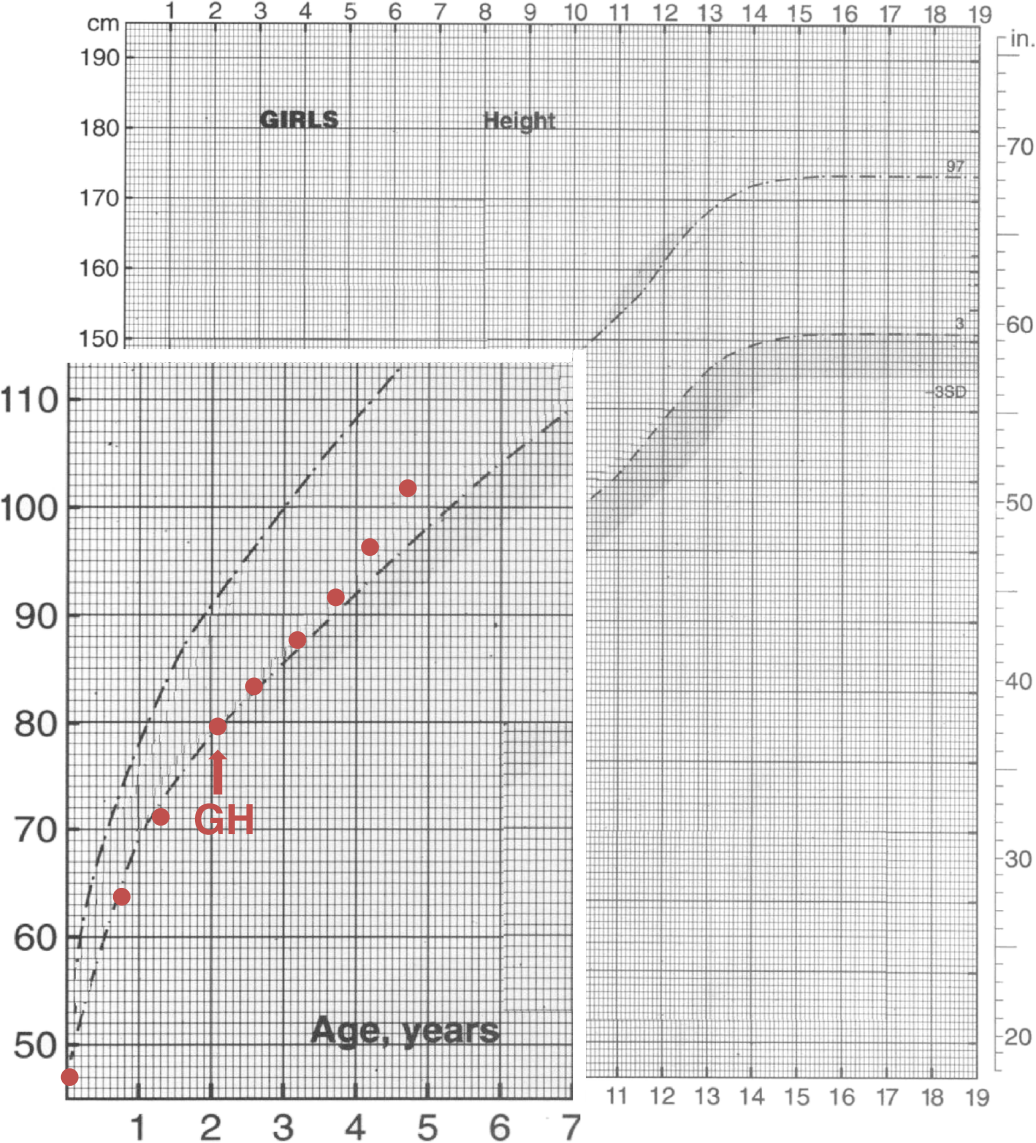
Height curve of the subject. The *arrow* indicates the start of GH substitutive therapy

At the age of 14 months, the patient presented a height of 70.5 cm (−2.19 SDS), a weight of 7320 g (BMI: −2.05 SDS), and a bone age of 8 months. At the age of 24 months, her length was 79 cm (−1.68 SDS) and weight 8300 (BMI −2.86 SDS). Therefore, because of the progressive growth failure, she underwent a careful endocrine evaluation that showed an insufficient GH response to two pharmacological stimuli (GH peaks: 4.3 ng/ml and 8 ng/ml; normal values >10 ng/ml). According to our national rules, arginine infusion was administered on two different days to avoid side effects due to the age and weight of the patient. Low serum insulin-like growth factor-I (IGF-I) value (38.7 ng/ml, −3.19 SDS) confirmed the diagnosis of GHD. Brain magnetic resonance image (MRI) documented a normal morphology; in particular, morphologically normal adenohypophysis, in place neurohypophysis keeping regular signal hyperintensity and aligned pituitary stalk. The replacement therapy with GH (0.21 mg/Kg/week divided in six weekly doses) was promptly started at 25 months of life. A significant increase in linear growth was observed during the first and the second year of therapy (growth rate: 8.04 cm/year, 0.19 SDS and 8.79 cm/year, 1.67 SDS, respectively), as shown in Fig. 1.

Furthermore, a recovery of the weight defect was observed already after the 1^st^ year of GH treatment, independently of food intake (age 3.1 years, height 87.6 cm, −1.66 SDS; weight 12 Kg, BMI −0.16 SDS).

## Discussion

Chromosome 22q11 microdeletion could be associated with DGS, VCFS [10], conotruncal anomaly face syndrome (CTAF) [11], autosomal dominant Optiz G/BBB [12] and Cayler Cardiofacial syndrome [13]. These syndromes are characterized by an enormous phenotypic heterogeneity. The most frequent feature is the association of congenital cardiac abnormalities and neonatal hypocalcaemia (more than 70 % of patients) which leads to the diagnosis in the first months of life [14]. Moreover, a thymus hypoplasia can be found in these subjects with consequent immunodeficiency (75 % of the patients) [10, 15]. Developmental delay, facial dysmorphia, palatal anomalies, feeding difficulties and other clinical features are also seen in most infants, but are less consistent and not easily recognized in children [15, 16]. Short stature is one of these features and has been related to a constitutional delay [7]. When multiple defects and/or features commonly seen in the syndrome are present simultaneously in a child, the decision to check 22q11.2 deletion is often straightforward. On the contrary, signs and symptoms might sometimes be subtle and could be easily overlooked, so that many patients remain undiagnosed.

Our patient had none of the characteristic clinical features of the syndrome. However, her progressive growth failure, which is typical in primitive dwarfism, led us to investigate the subject from a genetic point of view, using the array-CGH analysis. We found the 22q11.2 deletion, which could not be suspected on the basis of clinical symptoms alone. In infants showing progressive growth failure, we therefore suggest that a careful clinical and laboratory investigation to exclude congenital malformations and chronic diseases is mandatory. Then, after these conditions have been excluded, a genetic analysis to find chromosomal abnormalities responsible for failure to thrive is important, notwithstanding the lack of a suggestive family history and dysmorphic facial features. An early diagnosis of 22q11.2 syndrome is important in order to act on symptoms such as growth development or speech and language delay and to avoid conditions which may develop during adulthood, especially behavioural and psychiatric disorders [17]. In fact, patients with 22q11 deletion perform worse in neurocognitive tests such as verbal memory [18] and spatial working memory [19] after the onset of psychosis. Psychotic disorder is associated with a deterioration of social and academic skills [20] as well as a deficit of intelligence quotient (IQ).

Furthermore, once the diagnosis has been made in a child, it is necessary to extend the genetic investigation to the patient’s parents, although they are asymptomatic, because 10 % of the typical mutations of the syndrome are inherited from an affected parent [21]. It is therefore necessary to broaden the index of suspicion, particularly in the parents of affected probands, in order to identify mildly affected individuals with the 22q11.2 deletion which will ultimately lead to appropriate cognitive remediation, medical management, and recurrence risk counselling for these families. Clinicians should be aware that the deletion is more common than has been previously reported.

Moreover, this baby showed non-severe isolated GHD and promptly started GH substitutive treatment in order to improve her height and weight. The condition of GHD in patients with 22q11.2 microdeletion has rarely been reported [22] and was generally found in children who were significantly below the 5^th^ percentile for height [23]. No explanation was hypothesized to explain this association. Replacement therapy in GHD children with a 22q11.2 deletion induces a sustained improvement in height and growth velocity [22]. Therefore, in children with the 22q11.2 deletion and short stature/poor growth, GHD should be suspected.

## Conclusions

In conclusion, we report the case of a child who showed failure to thrive not associated with any dysmorphic feature, in whom deletion of chromosome 22q11.2 was found, suggesting the diagnosis of DiGeorge syndrome.

Since it is now being increasingly recognized that many patients with failure to thrive may have chromosomal abnormalities, we strongly believe that genetic analysis such as array-CGH in this field may improve the clinician’s ability to diagnose congenital causes of short stature. The identification of a molecular aetiology for short stature can end the diagnostic workup for a patient, provide the family with an answer as to why their child is not growing normally and alert the clinician to other medical comorbidities. Furthermore, determination of a particular genetic abnormality is invaluable for genetic counselling.

### Consent to publish

Written informed consent was obtained from the patient’s parents for publication of this Case report. A copy of the written consent is available for review by the Editor-in-Chief of this journal.

## Abbreviations

array-CGH: comparative genomic hybridization array
BMI: body mass index
DGS: DiGeorge syndrome
GH: growth hormone
GHD: growth hormone deficiency
IGF-I: insulin-like growth factor-I
SDS: standard deviation score
VCFS: velocardiofacial syndrome
